# Improved Elution Conditions for Native Co-Immunoprecipitation

**DOI:** 10.1371/journal.pone.0018218

**Published:** 2011-03-23

**Authors:** Robin Antrobus, Georg H. H. Borner

**Affiliations:** Cambridge Institute for Medical Research, Wellcome Trust, Addenbrooke's Hospital, University of Cambridge, Cambridge, United Kingdom; University of Minnesota, United States of America

## Abstract

**Background:**

Native immunoprecipitation followed by protein A-mediated recovery of the immuno-complex is a powerful tool to study protein-protein interactions. A limitation of this technique is the concomitant recovery of large amounts of immunoglobulin, which interferes with down-stream applications such as mass spectrometric analysis and Western blotting. Here we report a detergent-based “soft” elution protocol that allows effective recovery of immunoprecipitated antigen and binding partners, yet avoids elution of the bulk of the immunoglobulin.

**Methodology/Principal Findings:**

We assessed the performance of the soft elution protocol using immunoprecipitation of Adaptor protein complex 1 (AP-1) and associated proteins as a test case. Relative to conventional elution conditions, the novel protocol substantially improved the sensitivity of mass spectrometric identification of immunoprecipitated proteins from unfractionated solution digests. Averaging over three independent experiments, Mascot scores of identified AP-1 binding partners were increased by 39%. Conversely, the estimated amount of recovered immunoglobulin was reduced by 44%. We tested the protocol with five further antibodies derived from rabbit, mouse and goat. In each case we observed a significant reduction of co-eluting immunoglobulin.

**Conclusions/Significance:**

The soft elution protocol presented here shows superior performance compared to standard elution conditions for subsequent protein identification by mass spectrometry from solution digests. The method was developed for rabbit polyclonal antibodies, but also performed well with the tested goat and mouse antibodies. Hence we expect the soft elution protocol to be widely applicable.

## Introduction

Native immunoprecipitation (IP) is a widely-used tool in the study of protein-protein interactions. Standard protocols follow a regime of solubilizing cells in mild detergents, incubation with an antibody cross-reacting with the protein of interest, and recovery of the antibody-protein complex with protein A conjugated to an inert matrix, such as sepharose beads. The immunoprecipitated protein and any potential binding partners are eluted by boiling the matrix in SDS-containing buffer, which disrupts the protein A-immunoglobulin (Ig) interaction [1].

An inherent complication of this method is the co-elution of large amounts of Ig, which usually accounts for the majority of the recovered material. The Ig can cause problems for down-stream applications such as SDS-PAGE, where it may mask whole regions of the gel, or cause high background on Western blots. Furthermore, it can interfere with mass-spectrometric identification of low-abundance co-precipitated proteins, especially when the immunoprecipitated material is analyzed directly from unfractionated solution digests.

Co-elution of Ig can be avoided by covalent coupling of antibodies to the matrix, but this requires individual optimization of the coupling conditions to prevent loss of antibody-antigen binding, and is hence time-consuming and expensive. Here we report an alternative approach based on a “soft” elution protocol that allows recovery of the immunoprecipitated material, whilst leaving a substantial proportion of the Ig bound to the protein A matrix.

## Results and Discussion

### Developing improved elution conditions

SDS is a potent anionic detergent that very effectively disrupts protein-protein interactions [2]. We observed that at a concentration of 0.2% SDS (50 mM Tris, pH = 8.0) incubation at 25°C is already sufficient to elute rabbit polyclonal Ig from protein A sepharose beads (data not shown). To reduce the potency of the SDS, we titrated in small amounts of the non-ionic detergent Tween-20. We established that at 25°C, a mixture of 0.2% SDS and 0.1% Tween-20 leaves the protein A-Ig interaction mostly intact, but allows effective elution of immunoprecipitated antigens as well as antigen-associated proteins.

**Figure 1:**
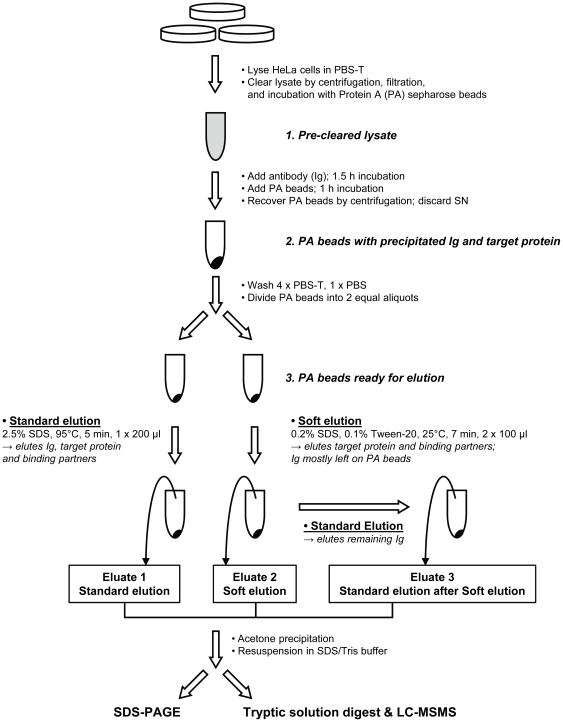
Graphical overview: Immunoprecipitation, standard vs improved elution protocol, and down-stream analysis. The diagram highlights key steps of the soft elution protocol, and shows how the comparison with standard elution conditions was performed. A complete description of the process can be found in File S1.

Based on these findings, we recommend the following elution conditions:

*The elution protocol assumes that a native immunoprecipitation in PBS-T (phosphate buffered saline + 1% Triton X-100) has been performed, using protein A sepharose beads to recover the immuno-complex (see File S1 for the complete protocol). A diagrammatic overview of the procedure is shown in Figure 1.*

### “Soft” Elution Protocol

1. Wash sepharose beads 4 times in PBS-T.

2. Wash beads once in PBS, to remove detergent.

3. Resuspend beads in 100 µL Soft Elution Buffer (0.2% (w/V) SDS, 0.1% (V/V) Tween-20, 50 mM Tris-HCl, pH = 8.0). Incubate for 7 min at 25°C, shaking at 1000 rpm (1.5 mL tube).

4. Pellet beads by centrifugation. Remove supernatant, and transfer to 1.5 mL collection tube.

6. Repeat elution (step 3) once.

7. Pellet beads by centrifugation. Remove supernatant, and pool with eluate from step 4.

8. Centrifuge pooled eluates at 16,000 x g for 1 min, to pellet carried-over beads. Transfer supernatant (∼200 µL) to fresh 1.5 ml tube.

9. Add 1 mL acetone (−20°C), and mix. Incubate at −20°C for 3–20 h to precipitate protein.

10. Centrifuge at 10,000 x g for 5 min, 4°C.

11. Remove supernatant. Air-dry pellet for 5 min.

12. Resuspend pellet in a buffer of your choice.

### Performance of the soft elution protocol

We assessed the performance of our soft elution protocol by using Adaptor protein-1 (AP-1) as a test case. AP-1 is a stable complex that consists of four subunits (γ, β1, µ1, σ1); the σ-subunit occurs in three isoforms [3]. Furthermore, AP-1 has several established binding partners, including aftiphilin, γ-synergin, p200, KIF13A, and p34 [4]–[6].

**Figure 2:**
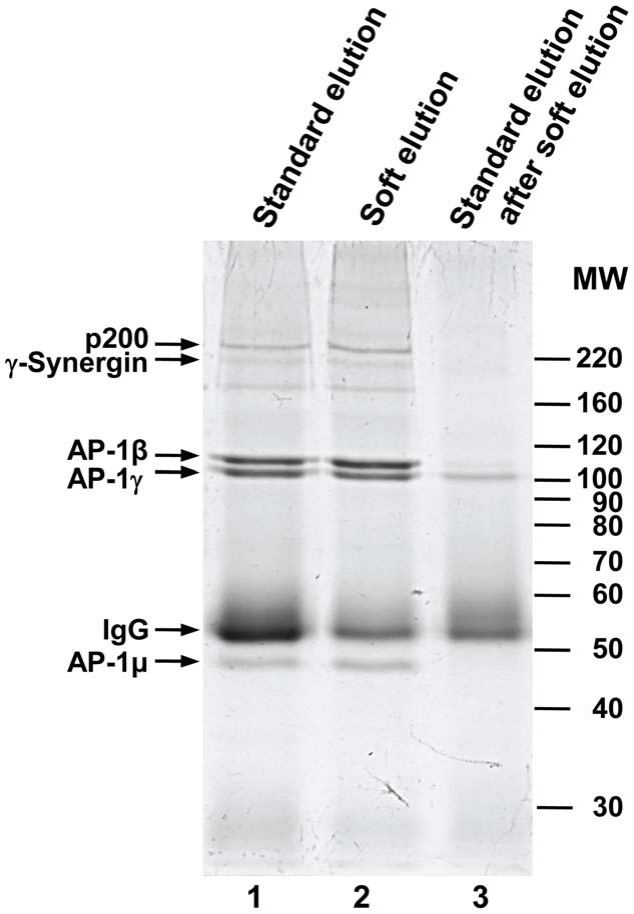
SDS-PAGE analysis of standard and improved elution protocols. Native immunoprecipitation of the AP-1γ subunit from HeLa cell lysates was performed as described in File S1. Immuno-complexes were recovered by addition of protein A sepharose beads. Prior to elution, beads were split into two equal aliquots. One was subjected to typical high SDS/high heat elution conditions (“standard elution”, lane 1), the other to our “soft” elution protocol (lane 2). Following soft elution, beads were re-eluted using the standard protocol (lane 3), to recover protein still bound to the beads. Thus, lane 3 shows the proportion of immunoglobulin (IgG) that is avoided through soft elution. Proteins in selected bands were identified by mass spectrometry (small arrows). For each band only the top scoring hit is shown. AP-1β, AP-1γ and AP-1µ are components of the AP-1 complex; p200 and γ-Synergin are known AP-1 associated proteins. IgG: rabbit Ig gamma chain C region. Approximate molecular weights are indicated (MW, in kD). Gels were stained with Coomassie G-250.

The AP-1γ-subunit was immunoprecipitated from detergent lysates of HeLa cells using a rabbit polyclonal antibody [7]. Protein A sepharose beads with bound immunoprecipitated material were split into two equal aliquots, and eluted in parallel under standard or improved conditions (Figure 1). Recovered protein was analyzed by SDS-PAGE (Figure 2). Soft elution allowed highly effective recovery of the antigen and associated proteins, whilst it substantially reduced the amount of co-eluting Ig compared to standard elution conditions.

To determine whether the reduction of Ig facilitates the identification of co-immunoprecipitated proteins, we again eluted AP-1γ IPs under standard or improved conditions in parallel. Eluates were subjected to tryptic solution digest, and analyzed by mass spectrometry. In three independent repeats, we identified ten AP-1 constituent and associated proteins with both elution methods. However, the AP-1σ1c subunit was detected in only one experiment under standard elution conditions, whereas it was identified in all three repeats using soft elution (Table 1). Hence, soft elution allowed the consistent identification of a minor co-precipitant that was largely missed under standard elution conditions.

**Table 1:** Mass spectrometric identifications from all three AP-1γ IPs.

Protein	Experiment 1				Experiment 2				Experiment 3			
	Score		Peptides		Score		Peptides		Score		Peptides	
	Soft	Std	Soft	Std	Soft	Std	Soft	Std	Soft	Std	Soft	Std
AP-1γ1	726	635	28	37	2757	1737	118	77	2582	3450	86	147
AP-1β	2072	1779	92	109	3792	2655	160	111	5854	4996	244	212
AP-1µ1a	495	1348	44	96	2906	1754	148	100	3124	3650	145	178
AP-1σ1a	901	1406	14	26	839	383	25	11	777	978	24	24
AP-1σ1b	74	62	5	6	141	68	9	3	162	224	15	11
AP-1σ1c	98	-	2	-	31	-	1	-	141	91	11	3
Aftiphilin	332	241	20	9	1235	519	34	20	2622	2644	88	83
γ-Synergin	1052	549	21	18	1068	404	29	16	5596	3881	188	138
p200	282	354	13	12	903	494	39	17	4116	4074	161	165
KIF13a	745	524	29	21	1386	851	59	30	280	191	12	11
p34	63	50	2	2	330	269	19	14	105	83	3	3

This table shows AP-1 constituents and associated proteins identified in three independent AP1-γ immunoprecipitations. In each experiment, samples (ie Protein A sepharose beads with bound antigen) were split into two equal aliquots prior to elution. Aliquots were either subjected to the soft elution protocol (“Soft”) reported in this study, or to standard elution conditions (“Std”). Eluates were subjected to tryptic solution digest without further fractionation, and analyzed by LC-MSMS. Mascot scores reflecting the confidence of identification [8], and the number of peptides identified for each protein are indicated. Please note that the AP-1σ1c subunit was not identified in experiments 1 and 2 using standard elution conditions.

Experiments 1 and 2 were performed in MES-D buffer; experiment 3 was performed in PBS-T buffer (see File S1 for details).

Next we investigated if the quality of mass spectrometric protein identification also benefited from the soft elution protocol. We compared Mascot scores, which reflect the confidence of protein identification [8], of the seven most abundant co-precipitants (Table 2). On average, scores were almost 40% higher in soft-eluted samples, demonstrating that the soft elution protocol significantly improved down-stream mass spectrometric analysis.

**Table 2:** Mass spectrometric analysis of AP-1γ IPs: relative performance of soft vs standard elution.

Protein	Mascot Scores Soft/Standard	Peptide Count Soft/Standard
AP-1β	1.25	1.15
AP-1µ1a	0.96	0.92
AP-1σ1a	1.21	1.27
Aftiphilin	1.58	1.66
γ-Synergin	2.00	1.45
p200	1.21	1.45
KIF13a	1.51	1.48
**Average ± SD**	**1.39±0.34**	**1.34±0.25**

For a meaningful comparison only the seven most abundant co-precipitants of AP-1γ were included (see Table 1). Ratios are the averages of three independent experiments.

**Figure 3:**
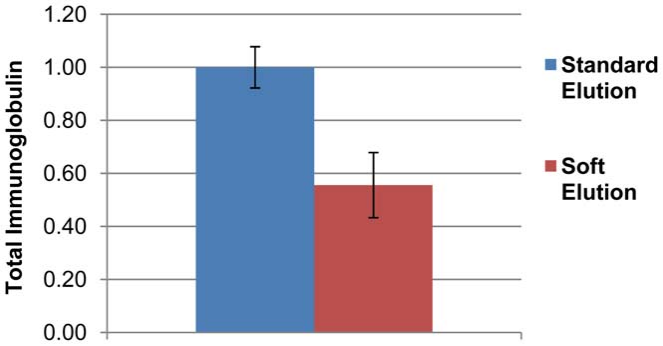
Relative abundance of immunoglobulin (Ig) in IP eluates. The amount of rabbit Ig present in IP eluates was estimated by summation of emPAI values. Average total Ig in standard eluates was set to 1, and average total Ig present in soft elution eluates was expressed as a fraction of 1. The figure shows the results of three independent experiments (error bars  =  SEM).

Although mass spectrometry is not intrinsically quantitative, the number of identified peptides can be converted into “emPAI” values (exponentially modified protein abundance index; [9]), an approximate measure of absolute protein abundance. We used emPAI values calculated by Mascot to estimate the amount of Ig present in standard and soft eluted samples. For each sample, we summed all Ig-related emPAI values to obtain a measure of total Ig present (Figure 3). Averaging across the three experiments, we observed a 44% reduction in Ig through soft elution, consistent with our SDS-PAGE analysis (Figure 2).

**Figure 4:**
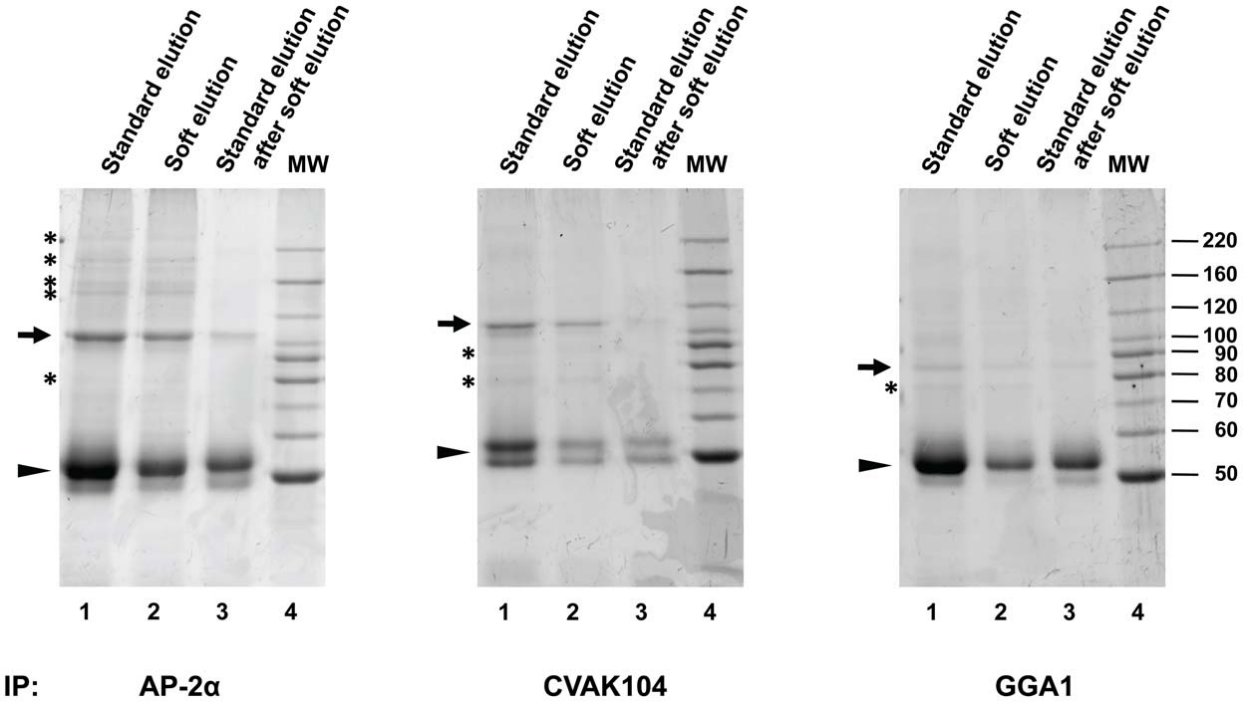
Performance of the improved elution protocol with various rabbit antibodies. Immunoprecipitations and SDS-PAGE were performed as in Figure 2, with the indicated antibodies (all rabbit polyclonal). Immunoprecipitated proteins were eluted from the protein A sepharose beads using standard conditions (lane 1), or soft-elution (lane 2). Soft-eluted beads were then subjected to standard elution conditions, to recover any remaining material (lane 3). Hence, lane 3 shows the proportion of immunoglobulin (Ig) avoided through soft elution. Lane 4 shows molecular weight markers (MW, in kD). Gels were stained with Coomassie G-250. Small arrows indicate the precipitated primary antigens (identified by molecular weight and through comparison with Western blots). Arrowheads indicate IgG heavy chain bands (identified by molecular weight and abundance). Asterisks indicate examples of proteins that co-precipitate with the primary antigen. The figure shows that soft elution substantially reduces the amount of co-eluting Ig for all tested antibodies, whilst allowing efficient recovery of co-precipitating proteins.

Next we investigated if the soft elution protocol also works for other rabbit polyclonal antibodies. We performed immunoprecipitations with antibodies against AP-2α [10], CVAK104 [11], and GGA1 [12], and as before eluted in parallel under standard or improved conditions (Figure 4). In all three cases, soft elution resulted in the effective recovery of co-precipitating proteins, as well as in a substantial reduction of co-eluting Ig.

**Figure 5:**
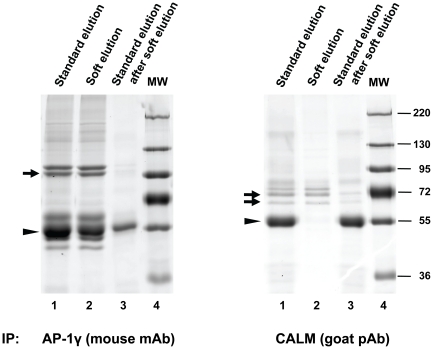
Performance of the improved elution protocol with mouse and goat antibodies. Immunoprecipitations and SDS-PAGE were performed as in Figures 2 and 4, with an IgG_2a_ mouse monoclonal antibody (mAb) against AP-1γ (left panel), or a goat polyclonal antibody (pAb) against CALM (right panel). Gels were stained with Coomassie G-250. Small arrows indicate the precipitated primary antigens (identified by molecular weight and through comparison with Western blots). Please note that CALM occurs in two isoforms (62 kD and 72 kD). Arrowheads indicate IgG heavy chain bands (identified by molecular weight and abundance). The figure shows how the soft elution protocol performs with non-rabbit antibodies. In case of the goat polyclonal antibody, co-elution of Ig is almost completely avoided through soft elution.

Antibodies derived from different species vary in their affinity for protein A [13], and this may affect the performance of the soft elution protocol. To test this, we performed immunoprecipitations with a mouse monoclonal antibody against AP-1γ (mAb100/3, an IgG_2a_), and a goat polyclonal antibody against CALM (clathrin assembly lymphoid myeloid leukemia protein [14]). While protein A has relatively high affinity for mouse IgG_2a_, it binds only weakly to polyclonal goat Ig [13]. Hence, we used protein G sepharose to recover CALM immuno-complexes. The results are shown in Figure 5. In the case of the mouse antibody, soft elution allowed some reduction of recovered Ig; the relative difference to standard elution was however not as pronounced as that observed for rabbit antibodies (Figures 2 and 4). Remarkably, for the goat polyclonal antibody soft elution allowed almost complete avoidance of Ig co-elution; nearly all the Ig was left on the protein G beads, while the antigen was effectively recovered. These data suggest that the soft elution protocol is suitable for antibodies derived from species other then rabbit.

### Conclusion and Perspective

The IP soft elution protocol presented here shows superior performance compared to standard elution conditions for subsequent protein identification by mass spectrometry from solution digests. We have formally demonstrated this for AP-1 IPs (Figures 2&3, Tables 1&2). Since we used a polyclonal antibody (ie a diverse mixture of antibodies with a range of affinities), our method will be widely applicable. Indeed, we have tested the protocol with further rabbit polyclonal antibodies (Figure 4), and in all cases observed a similar reduction of Ig relative to standard elution conditions as seen in Figure 2. Furthermore, soft elution appears to be suitable for use with mouse IgG_2a_ monoclonal antibodies, and with a minor modification (using protein G instead of protein A), it shows excellent performance with goat polyclonal antibodies (Figure 5). Hence we expect our method to work for most rabbit polyclonal antibodies, as well as other antibodies with high affinity for protein A or protein G.

## Materials and Methods

A detailed version of the IP-soft elution protocol as well as a full description of the mass spectrometric analyses performed in this study can be found in File S1. All LC-MSMS data presented here were generated using a nanoACQUITY LC (Waters) coupled to an LTQ OrbiTrap XL mass spectrometer (Thermo).

### Antibodies and reagents

Immunoprecipitations were performed with the following antibodies: AP-1γ rabbit polyclonal antibody [7]; AP-2α [10]; CVAK104 [11]; GGA1 [12]; AP-1γ mouse monoclonal antibody (mAb100/3, Sigma-Aldrich); CALM (C-18, Santa Cruz Biotechnology). Detergent lysates were prepared from HeLaM cells [15]. Protein A sepharose (#17-0780-01) and protein G sepharose (#17-061801) were obtained from GE Healthcare. Chemicals were purchased from Sigma-Aldrich.

### Gel analysis

SDS-PAGE was performed according to a standard protocol [13]. Gels were stained with Coomassie G-250 SimplyBlue SafeStain (Invitrogen, #LC6060), and scanned with an Odyssey Infrared Imager (LI-COR Biosciences). Scans were exported as TIFFs. Adjustment of brightness and contrast as well as despeckling was performed in Adobe Photoshop.

### Application notes

The soft elution protocol has been thoroughly tested with rabbit, mouse (IgG_2A_), and goat antibodies. It is likely that it will perform well with antibodies derived from other species, provided they interact strongly with protein A or protein G. When using the soft elution protocol with an untested class of antibodies, it is recommended to select the protein with the highest predicted affinity (see for example http://www.piercenet.com/files/TR0034-Ab-binding-proteins.pdf).

The IP soft elution protocol was primarily designed to facilitate the mass spectrometric identification of proteins co-precipitating with the primary antigen. We have tested the protocol with a wide range of antibodies, and in most cases observed a small reduction in the amount of recovered primary antigen (eg Figure 2, faint AP-1γ band in lane 3). Our data suggest that this reduction is not detrimental for the purpose of protein identification (Table 1 – average Mascot scores for AP-1γ are still higher in soft eluted samples). Nevertheless, in cases where complete recovery of the primary antigen is desired, standard elution protocols may be more suitable. For pilot experiments, soft and standard elutions can be performed sequentially (as shown in lanes 2 and 3 of Figure 2), to ensure recovery of all immunoprecipitated material.

## Supporting Information

File S1 Complete protocol for native immunoprecipitation and soft elution.
(DOC)

## References

[1] Bonifacino JS, Dell'Angelica EC, Springer TA (2006). Immunoprecipitation.. Curr Protoc Neurosci.

[2] Helenius A, Simons K (1975). Solubilization of membranes by detergents.. Biochim Biophys Acta.

[3] Boehm M, Bonifacino JS (2001). Adaptins: the final recount.. Mol Biol Cell.

[4] Hirst J, Borner GHH, Harbour M, Robinson MS (2005). The aftiphilin/p200/gamma-synergin complex.. Mol Biol Cell.

[5] Delevoye C, Hurbain I, Tenza D, Sibarita JB, Uzan-Gafsou S (2009). AP-1 and KIF13A coordinate endosomal sorting and positioning during melanosome biogenesis.. J Cell Biol.

[6] Page LJ, Sowerby PJ, Lui WW, Robinson MS (1999). Gamma-synergin: an EH domain-containing protein that interacts with gamma-adaptin.. J Cell Biol.

[7] Seaman MN, Sowerby PJ, Robinson MS (1996). Cytosolic and membrane-associated proteins involved in the recruitment of AP-1 adaptors onto the trans-Golgi network.. J Biol Chem.

[8] Perkins DN, Pappin DJ, Creasy DM, Cottrell JS (1999). Probability-based protein identification by searching sequence databases using mass spectrometry data.. Electrophoresis.

[9] Ishihama Y, Oda Y, Tabata T, Sato T, Nagasu T (2005). Exponentially modified protein abundance index (emPAI) for estimation of absolute protein amount in proteomics by the number of sequenced peptides per protein.. Mol Cell Proteomics.

[10] Ball CL, Hunt SP, Robinson MS (1995). Expression and localization of alpha-adaptin isoforms.. J Cell Sci.

[11] Borner GHH, Rana AA, Forster R, Harbour M, Smith JC (2007). CVAK104 is a novel regulator of clathrin-mediated SNARE sorting.. Traffic.

[12] Hirst J, Lui WW, Bright NA, Totty N, Seaman MN (2000). A family of proteins with gamma-adaptin and VHS domains that facilitate trafficking between the trans-Golgi network and the vacuole/lysosome.. J Cell Biol.

[13] Sambrook J, Russel DW (2001). Molecular Cloning: A Laboratory Manual.Third Edition..

[14] Tebar F, Bohlander SK, Sorkin A (1999). Clathrin assembly lymphoid myeloid leukemia (CALM) protein: Localization in endocytic-coated pits, interactions with clathrin, and the impact of overexpression on clathrin-mediated traffic.. Mol Biol Cell.

[15] Tiwari RK, Kusari J, Sen GC (1987). Functional equivalents of interferon-mediated signals needed for induction of an mRNA can be generated by double-stranded RNA and growth factors.. EMBO J.

