# Supplementary material for: Improved Elution Conditions for Native Co-Immunoprecipitation
Source: PLoS One. 2011 Mar 23;6(3):e18218. doi: 10.1371/journal.pone.0018218 (PMC3063181; doi:10.1371/journal.pone.0018218)
Supplement: File S1 — Complete protocol for native immunoprecipitation and soft elution. (DOC) [file pone.0018218.s001.doc]

**Online Supplemental Materials and Methods**

**Complete protocol for native immunoprecipitation and soft elution**

All operations are carried out at 4°C, unless specified otherwise.

**Reagents**

PBS (“Phosphate buffered saline”: NaCl 137 mM; KCl 2.7 mM; Na_2_HPO_4_ 10 mM; KH_2_PO_4_ 1.76 mM; pH = 7.4)

PBS-T (PBS adjusted to 1% Triton X-100, using a 10% Triton stock (Sigma-Aldrich, #93443))

Soft Elution Buffer (0.2% (w/V) SDS (Sigma-Aldrich, #71736), 0.1% (V/V) Tween-20 (Sigma-Aldrich, #P1379), 50 mM Tris-HCl (Sigma-Aldrich, #T1503), pH = 8.0)

1 x Sample buffer (2% (w/V) SDS, 10% (V/V) glycerol, 2% (V/V) β-mercaptoethanol, trace of bromophenol blue, 50 mM Tris-HCl, pH = 6.8)

Acetone (-20°C)

Protein A (PA) sepharose CL-4B beads (GE Healthcare, #17-0780-01)

**Preparation of cell lysate**

1. Rinse HeLa cells (500 cm^2^, confluent monolayer) twice with ice-cold PBS.

2. Using a cell scraper, resuspend cells in 5 mL PBS-T, and extract for 20 min, rotating (8 rpm), in a 15 mL tube.

3. Centrifuge lysate at 4,000 x g for 3 min to pellet cell debris. Discard pellet.

4. Centrifuge lysate at 200,000 x g for 20 min (MLA-80 rotor, Beckman). Discard pellet.

5. Pass lysate through a syringe filter (0.2 μm).

6. To pre-clear lysate, add 50 μL PA sepharose bead slurry (50% V/V in PBS), and incubate for 30 min, rotating at 8 rpm.

7. Centrifuge lysate at 2,200 x g for 3 min to pellet beads; transfer supernatant (~ 5 mL) to a fresh tube. This is the starting material for the IP. Protein concentration should be 2-4 mg/mL.

**Immunoprecipitation**

8. Add rabbit polyclonal antibody to the lysate. Useful concentration is highly antibody dependent, and requires individual optimization. Recommended starting point: 2 to 5-fold higher than used for Western blotting.

9. Incubate for 1h 30 min, rotating (8 rpm).

10. Add 50 μL PA sepharose bead slurry (50% V/V in PBS), and incubate for 1h, rotating at 8 rpm.

11. Centrifuge at 2,200 x g for 3 min to pellet beads. Discard supernatant.

**Washing**

12. Wash beads once in 5 mL PBS-T. Centrifuge at 2,200 x g for 3 min to pellet beads.

13. Resuspend beads in 1 mL of PBS-T, and transfer to 1.5 mL tube. Centrifuge at 2,300 x g for 20 s to pellet beads.

14. Wash beads twice more by resuspending in 1 mL PBS-T and centrifugation as in step 13.

15. Wash beads once in 1 mL PBS only, to remove detergent.

**Soft elution and recovery**

16. Resuspend beads in 100 μL Soft Elution Buffer. Incubate for 7 min at 25°C, shaking at 1000 rpm (to prevent beads from settling).

17. Centrifuge at 2,300 x g for 20 s (20-25°C) to pellet beads. Carefully remove supernatant, and transfer to 1.5 mL collection tube.

18. Repeat step 16.

19. Centrifuge at 16,000 x g for 1 min (20-25°C) to pellet beads. Carefully remove supernatant, and pool with eluate from step 17.

20. Centrifuge eluate at 16,000 x g for 1 min (20-25°C), to pellet carried-over beads. Transfer supernatant (~200 μL) to fresh 1.5 mL tube.

21. Add 1 mL acetone (-20°C), and mix. Incubate at -20°C for 3-20 h to precipitate protein.

22. Centrifuge at 10,000 x g for 5 min, 4°C. Remove supernatant. Air-dry pellet for 5 min.

23. Resuspend pellet in a buffer of choice (eg 1 x Sample buffer for SDS-PAGE).

**Variant protocol**

The protocol can be adapted for the use of other buffer systems. We have tested it for MES-D buffer (0.1 M 4-Morpholineethanesulfonic acid (MES); 1% (w/V) digitonin; 0.5 mM EGTA; 1 mM Mg(CH_3_COO)_2_; pH = 6.5), with equally good results. We recommend the same protocol as described above, using MES-D instead of PBS-T, and MES instead of PBS. MES-D is more likely to preserve weak protein-protein interactions, whereas PBS-T extracts membrane proteins more effectively.

MES-D is prepared as follows:

1. Add 0.4 g digitonin (Sigma-Aldrich, #D5628) and 10 mL H_2_O to a 50 mL tube.

2. Incubate at 99°C for 10 min to dissolve digitonin; vortex occasionally. Solution will remain turbid.

3. Allow to cool a little; add 8 mL 0.5 M MES (pH = 6.5, adjusted with NaOH).

4. Add 2 mL 10 mM EGTA.

5. Add 0.4 mL 100 mM Mg(CH_3_COO)_2_.

6. Add ~19 mL H_2_O to 40 mL final volume. Chill to 4°C.

7. Owing to the poor water-solubility of digitonin, MES-D requires filtering (0.2 μm syringe filter) immediately prior to use. Filtered MES-D should be a clear solution. If visible precipitation of digitonin occurs at any point, re-filter MES-D.

**Application notes**

The soft elution protocol has been thoroughly tested with rabbit, mouse (IgG_2A_), and goat antibodies. It is likely that it will perform well with antibodies derived from other species, provided they interact strongly with protein A or protein G. When using the soft elution protocol with an untested class of antibodies, it is recommended to select the protein with the highest predicted affinity (see for example <http://www.piercenet.com/files/TR0034-Ab-binding-proteins.pdf>).

The IP soft elution protocol was primarily designed to facilitate the mass spectrometric identification of proteins co-precipitating with the primary antigen. We have tested the protocol with a wide range of antibodies, and in most cases observed a small reduction in the amount of recovered primary antigen (eg Figure 2, faint AP-1γ band in lane 3). Our data suggest that this reduction is not detrimental for the purpose of protein identification (Table 1 – average Mascot scores for AP-1γ are still higher in soft eluted samples). Nevertheless, in cases where complete recovery of the primary antigen is desired, standard elution protocols may be more suitable. For pilot experiments, soft and standard elutions can be performed sequentially (as shown in lanes 2 and 3 of Figure 2), to ensure recovery of all immunoprecipitated material.

**Identification of proteins by mass spectrometry**

**Materials**

SDT; 4% (w/V) SDS, 100 mM DTT, 100 mM Tris-HCl pH 7.6

UA; 8 M urea, 100 mM Tris-HCl pH = 8.5

Alkylation solution; 50 mM IAA (iodoacetamide) in UA

ABC; 50 mM NH_4_HCO_3_

FASP digestion solution; modified Trypsin at 6 ng/μL in 50 mM NH_4_HCO_3_

FASP digestion wash; 0.5 M NaCl

Microcon YM-10 (Millipore, #42406)

StageTip solution A; 0.5% (V/V) acetic acid

StageTip solution B; 0.5% (V/V) acetic acid, 80% (V/V) acetonitrile

MS solvent; 0.1% (V/V) TFA

In-gel destain solution; 1:1 (V/V) H_2_O : acetonitrile

In-gel reduction solution; 10 mM DTT, 100 mM NH_4_HCO_3_

In-gel alkylation solution; 55 mM iodoacetamide, 100 mM NH_4_HCO_3_

In-gel digestion solution; 12.5 ng/µL modified trypsin, 50 mM NH_4_HCO_3_

In-gel peptide extraction solution 1; 1:1 (V/V) 50 mM NH_4_HCO_3_/acetonitrile

In-gel peptide extraction solution 2; 1:1 (V/V) 5% (V/V) formic acid/acetonitrile

**FASP solution digest [1]**

1. Resuspend pellet (from step 23 of the IP protocol) in a maximum of 30 µL SDT.
2. Mix sample with 200 µL UA in Microcon filter unit.
3. Centrifuge at 14,000 x g for 40 min. Discard flow through.
4. Repeat steps 2-3.
5. Add 100 µL alkylation solution to sample. Mix at 600 rpm for 1 min and incubate at RT for a further 5 min without mixing.
6. Centrifuge at 14,000 x g for 30 min. Discard flow through.
7. Add 100 µL ABC and centrifuge at 14,000 x g for 40 min.
8. Repeat step 7 twice.
9. Transfer the filter unit to a new collection tube.
10. Add 40 µL FASP digestion solution. Mix at 600 rpm for 1 min.
11. Incubate the filter unit at RT overnight in a wet chamber.
12. Centrifuge at 14,000 x g for 20 min.
13. Add 50 µL FASP digestion wash solution.
14. Centrifuge at 14,000 x g for 20 min.
15. Acidify pooled eluates for StageTip desalting.

**StageTip concentration and desalting [2]**

For steps 1 to 6 samples are reverse loaded and eluted by centrifugation at 300 x g. From step 7 onwards solvents are reverse loaded and samples are eluted using an Eppendorf CombiTip syringe.

1. Wet StageTip using 20 µL MeOH.
2. Wash with 20 µL StageTip solution B.
3. Equilibrate with 20 µL StageTip solution A.
4. Acidify sample using 50% (V/V) acetic acid until pH is approximately equal to solution A alone.
5. Load sample onto StageTip.
6. Wash sample with 20 µL StageTip solution A.
7. Elute sample using 10 µL StageTip solution B.
8. Repeat step 7 and pool eluates.
9. Evaporate sample almost to completion using a vacuum centrifuge**.**
10. Re-suspend sample in 10 µL MS solvent.

**In-gel digestion [3]**

Samples were analyzed by SDS-PAGE and stained with Coomassie blue. Bands of interest were manually excised and processed as follows:

1. Gel pieces are destained at room temperature for 15 min with shaking at 800 rpm using In-gel destain solution. Multiple washes may be necessary to fully destain samples**.**
2. Destained samples are equilibrated in 100 mM NH_4_HCO_3_, after 5 min equilibration an equal volume of acetonitrile is added and incubated at room temperature for 15 min with shaking at 800 rpm.
3. Equilibration solution is removed and replaced with In-gel reduction solution. Samples are reduced at 56°C for 45 min with shaking at 800 rpm.
4. In-gel reduction solution is removed and replaced with an equal volume of In-gel alkylation solution. Samples are alkylated for 30 min at room temperature in the dark with shaking at 800 rpm.
5. Samples are washed twice with 100 mM NH_4_HCO_3_ followed by equal volumes of acetonitrile as in step 2. Following washes, gel pieces are dehydrated and dried using acetonitrile and a vacuum centrifuge**.**
6. Sufficient In-gel digestion solution is added to cover the gel pieces and samples are incubated for 30 min on ice to allow gel pieces to rehydrate. Excess In-gel digestion solution is removed from the rehydrated gel pieces and replaced with fresh 50 mM NH_4_HCO_3_. Samples are digested overnight at room temperature**.**
7. Digested peptides are extracted once with In-gel peptide extraction solution 1 and twice with In-gel peptide extraction solution 2. All extraction steps are performed at room temperature with shaking at 800 rpm. The three washes are pooled in a 0.6 mL Maxymum Recovery microtube (Axygen, #MCT-060-L-C) and dried almost to completion using a vacuum centrifuge**.**
8. Resuspend sample in 10 µL MS solvent.

**LC-MSMS**

LC-MSMS data were generated using a nanoACQUITY LC (Waters, USA) coupled to an LTQ OrbiTrap XL mass spectrometer (Thermo, USA). Peptides were trapped on a 2 cm Symmetry 5µm C18 Trap column and eluted to a 25 cm 1.7 µm UPLC column (Waters, USA). Data were acquired in a DDA fashion with MS scans in the OrbiTrap at 60,000 (fwhm at 400 m/z) and parallel MSMS scans in the LTQ. Raw files were processed using MaxQuant [4] to generate .msm files which were searched against an in-house SwissProt database (version 57.1) using Mascot Daemon 2.2.2. Data were searched allowing a MS tolerance of 7 ppm and a MSMS tolerance of 0.5 Da. CAM cysteine was defined as a fixed modification while oxidized methionine, acetyl (protein N-terminus), and deamidated N/Q were defined as potential variable modifications. Peptides with ion scores < 30 were excluded and the significance threshold was set to p<0.01.

**Additional experimental information: parallel soft and standard elution from AP-1 IPs**

The AP-1 IPs reported in this study were performed as described above, scaled up two-fold. During the final wash step, the slurry of resuspended sepharose beads (~1 mL) was split into 2 x ~500 µL before the final pelleting step, to obtain two equal aliquots of PA beads. One aliquot was incubated in 200 μL of 2.5% (w/V) SDS, 50 mM Tris, pH = 8.0, at 95°C for 5 minutes (“standard elution conditions”). The other aliquot was first subjected to soft elution; to elute any remaining protein, the beads were then subjected to “standard elution conditions”. All eluates were precipitated with acetone over night. Recovered protein pellets were redissolved in 1 x Sample buffer, and incubated at 95°C for 5 min prior to SDS-PAGE.

**Supplemental References**

1. Wiśniewski JR, Zougman A, Nagaraj N, Mann M (2009) Universal sample preparation method for proteome analysis. Nat Methods 6: 359-362.

2. Rappsilber J, Mann M, Ishihama Y (2007) Protocol for micro-purification, enrichment, pre-fractionation and storage of peptides for proteomics using StageTips. Nat Protoc 2: 1896-1906.

3. Shevchenko A, Tomas H, Havlis J, Olsen JV, Mann M (2006) In-gel digestion for mass spectrometric characterization of proteins and proteomes. Nat Protoc 1: 2856-2860.

4. Cox J, Mann M (2008) MaxQuant enables high peptide identification rates, individualized p.p.b.-range mass accuracies and proteome-wide protein quantification. Nat Biotechnol 26: 1367-1372.
